# Mechanisms of infection by SARS-CoV-2, inflammation and potential links with the microbiome

**DOI:** 10.2217/fvl-2020-0310

**Published:** 2021-01-13

**Authors:** María Magdalena Aguirre García, Javier Mancilla-Galindo, Mercedes Paredes-Paredes, Álvaro Zamudio Tiburcio, Nydia Ávila-Vanzzini

**Affiliations:** 1 ^1^División de Investigación, Facultad de Medicina, Unidad de Investigación UNAM-INC, Instituto Nacional de Cardiología Ignacio Chávez, Juan Badiano No. 1, Col. Sección XVI, Tlalpan C.P. 14080, Ciudad de México, Mexico; 2 ^2^Departamento de Gastroenterología, Unidad de Trasplante de Microbiota Intestinal, Especialidades Médicas Nápoles, Oficina 12, Pennsylvania No. 209 Esq. Kansas, Col. Nápoles, Benito Juárez C.P 03810, Ciudad de México, Mexico; 3 ^3^Departamento de Consulta Externa, Instituto Nacional de Cardiología Ignacio Chávez, Juan Badiano No. 1, Col. Sección XVI, Tlalpan C.P. 14080, Ciudad de México, Mexico

**Keywords:** COVID-19, emerging diseases, immunology, infectious diseases, inflammation, microbe–host interaction, microbiome, miRNA, pandemic, SARS-CoV-2

## Abstract

The pandemic SARS coronavirus 2 utilizes efficient mechanisms to establish infection and evade the immune system. Established infection leads to severe inflammation in susceptible patients, the main hallmark of progression to severe coronavirus disease (COVID-19). Knowledge of the mechanisms of disease has expanded rapidly. As inflammation emerges as the central pathophysiological feature in COVID-19, elucidating how the immune system, lungs and gut communicate and interact with microbial components of the ecological niches that conform the human microbiome will shed light on how inflammation and disease progression are promoted. Studying the microbiome in COVID-19 could allow scientists to identify novel approaches to prevent severe inflammation by targeting components of the human microbiome. Innovation in the aforementioned is needed to combat this pandemic.

Human coronaviruses (HCoVs) were first isolated from patients with the common cold in the 1960s [1–3]. Seven HCoVs known to cause disease in humans have since been identified: HCoV-229E, HCoV-NL63, HCoV-OC43, HCoV-HKU1, the SARS coronavirus (SARS-CoV), the Middle East respiratory syndrome coronavirus and the novel SARS-CoV-2 [4]. The latter was identified after a spike in cases of pneumonia of unknown etiology in Wuhan, Hubei Province, China during December 2019 and was initially named novel coronavirus (2019-nCoV) [5,6]. The virus was renamed SARS-CoV-2 according to the International Committee on Taxonomy of Viruses classification criteria due to its genomic closeness to SARS-CoV; the disease caused by this virus was named coronavirus disease (COVID-19) according to the WHO criteria for naming emerging diseases [7]. SARS-CoV-2 belongs to the genera *Betacoronavirus* and shares a different degree of genomic similarity with the other two epidemic coronaviruses: SARS-CoV (∼79%) and Middle East respiratory syndrome coronavirus (∼50%) [8].

COVID-19 has caused considerable morbidity and mortality worldwide and has become the central phenomenon that is shaping our current societies. Human-to-human transmission is the main route of spread of the virus, mainly through direct contact, respiratory droplets and aerosols [9–12]. Management of COVID-19 has been extremely challenging due to its high infectivity, lack of effective therapeutics and potentially small groups of individuals (i.e., asymptomatic or mild disease) rapidly spreading the disease [13–17]. Although research describing COVID-19 and the mechanisms of infection by SARS-CoV-2 and its pathogenesis has expanded rapidly, there is still much to be learnt. Important gaps in knowledge which remain to be elucidated are the dynamic and complex interactions between the virus and the host’s immune system, as well as the potential interspecies communications occurring between ecological niches encompassing distinct microorganisms in both healthy individuals and persons living with chronic diseases, and how these interactions could determine or modulate disease progression and outcomes.

In this review, we describe recent insights into these topics, as well as remaining questions whose answers will allow us to understand how interactions between the virus, the immune system and microbial components could possibly be related to disease states in patients with COVID-19, as well as existing studies of the microbiome in patients with COVID-19.

## SARS-CoV-2 utilizes efficient mechanisms to establish infection & evade the innate immune response

The human angiotensin-converting enzyme 2 (hACE2) receptor has extensively been shown to be the primary receptor for SARS-CoV-2 through recognition of its ‘spike’ (S) glycoprotein, with subsequent priming by the transmembrane serine protease 2 and lysosomal cathepsins [5,18–21]. This surface protein has a novel furin-cleavage site between the S1 and S2 subunits [18], which resembles a homologous domain in the human epithelial sodium channel α-subunit [22]. Since the SARS-CoV-2 S protein is highly glycosylated and remains mostly in a closed prefusion conformation [23], pre-activation of the S protein by furin protease is thought to be an essential step to expose its receptor binding domain [21,24]. The pathogenesis of SARS-CoV-2 infection has been extensively reviewed elsewhere [25].

The hACE2 receptor has a high expression in the small intestine, testis, kidneys, heart, thyroid and adipose tissue; medium expression, in the lungs and liver; and low-to-no expression, in most cells and organs of the immune system (blood cells, spleen, bone marrow and blood vessels [unspecified]) [26]. Even when similar replication of SARS-CoV and SARS-CoV-2 has been found in the lower respiratory tract [27], SARS-CoV-2 replicates 100-fold more efficiently under the conditions encountered in the upper respiratory tract (i.e., 33°C) [28].

The interferon (IFN) response includes type I (IFN-α, IFN-β, IFN-ε, IFN-κ, IFN-ω), type II (IFN-γ) and type III (IFN-λ) IFNs, with type I and III IFNs being the first major line of defense against viruses [29]. The upper respiratory tract elicits greater type I and III IFN responses after viral infection when compared with bronchial epithelial cells [30]. However, SARS-CoV-2 induces only suboptimal early expression of type I, II and III IFN [27].

A highly efficient mechanism of entry to the cells, which involves the S protein remaining most of the time in a conformation which allows antigen occultation, with subsequent pre-activation of its receptor binding domain by furin before binding to hACE2 would explain why SARS-CoV-2 is highly successful at evading the innate immune system while achieving early accelerated replication. Other potential mechanisms of evasion of the innate immune system by SARS-CoV-2 have been reviewed elsewhere [31].

## Immune-mediated injury is the predominant pathophysiological driver in severe & critical COVID-19

**Figure 1.**
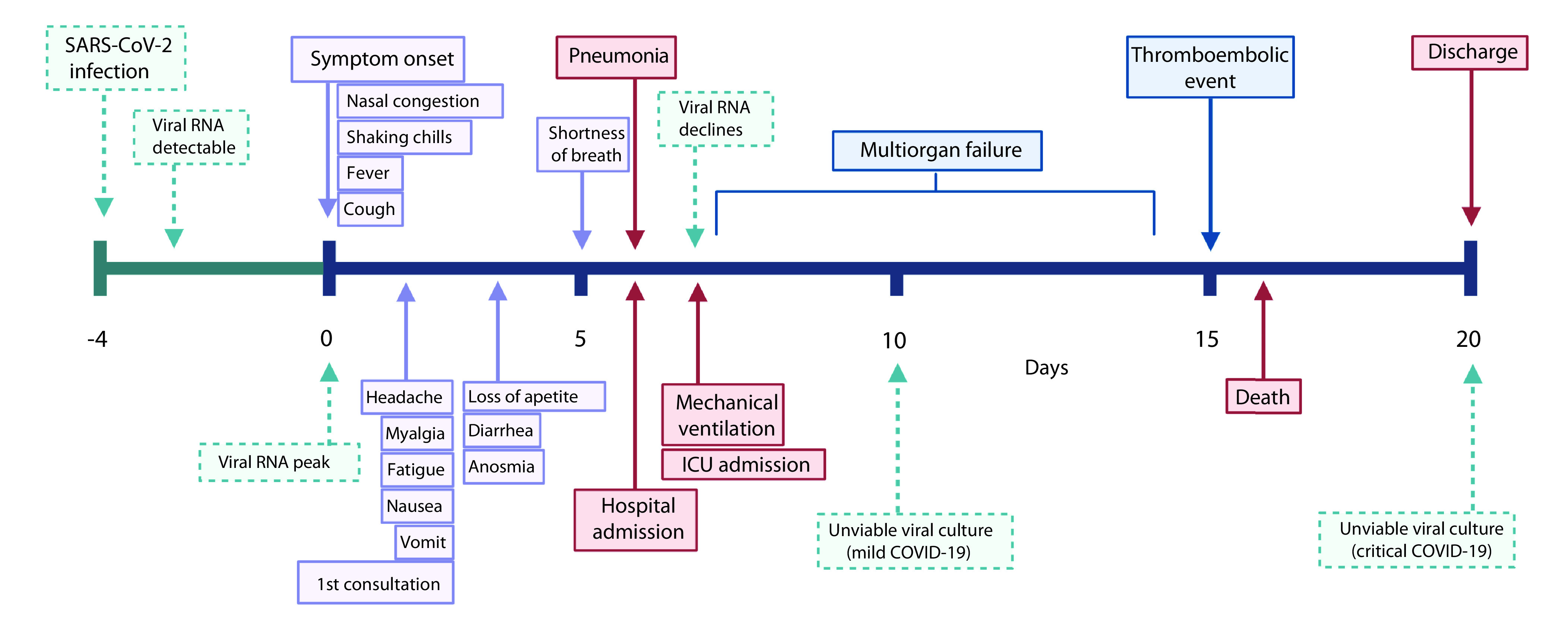
Time from onset of symptoms to specific events of interest in patients with symptomatic coronavirus disease. This timeline describes the time (mean days) at which different outcomes and other events of interest (solid boxes) occur in patients with COVID-19, including those who either experience mild-to-moderate disease or progress to severe-to-critical COVID-19. Events related to the dynamics of the virus (dashed boxes) include onset of infection, incubation period (dashed horizontal line), detection of viral ribonucleic acid (RNA) in respiratory specimens and the last day at which viable viral cultures have been obtained from patients with COVID-19. Figure created with BioRender.com. COVID-19: Coronavirus disease.

Even though many of the symptoms of COVID-19 could partly be attributed to viral tropism as mentioned earlier, a subset of clinical features is apparently due to immune-mediated mechanisms. Namely, thromboembolic events and multiorgan failure are considered complications of COVID-19 rather than direct effects of the virus, which only patients who progress to severe and critical COVID-19 develop. The delimitation between clinical manifestations caused predominantly by the virus and the complications due to immune dysregulation is evident since most symptoms and pneumonia have their onset in the first 6 days after symptom onset in most patients, whereas complications start to occur between days 7–8, coinciding with the time viral RNA starts to decline (Figure 1) [32–36].

It has been hypothesized that SARS-CoV-2 could disseminate to other organs via secondary viremia, targeting susceptible organs and causing direct damage [13] after successful replication in cells of the respiratory tract and evasion of innate immune defenses. However, damage to different organs is likely immune mediated as supported by the characteristic inflammatory storm described in patients who progress to severe and critical COVID-19 [37,38]. Two distinctive features have been noticed in these patients: progressive increases in inflammation and an unusual hypercoagulable state. Determinants of progression to an unregulated inflammatory state have not been completely elucidated yet, although the clinical characteristics of these patients and recent experimental studies have started to uncover some of the key elements.

Progression to severe and critical COVID-19 is known to occur more frequently in patients with increasing ages and significant comorbidities, most of which involve some degree of dysregulation of the renin–angiotensin–aldosterone system (RAAS), inflammation or both [39–43]. An adequate early immune response and optimal regulation of RAAS could be the key early features that prevent progression.

Type I and II IFNs induce greater expression of the hACE2 receptor in epithelial surfaces, and patients with inflammatory states involving upregulated IFNs (including COVID-19) are known to express high levels of hACE2 [44–46]. High basal expression of hACE2 in patients susceptible to COVID-19 could initially put them at increased risk through greater availability of hACE2. However, increased expression of hACE2 following robust early IFN responses in both healthy and diseased individuals would be important to guarantee proper regulation of angiotensin II, which is thought to be an important element in initiating and perpetuating the hyperinflammatory state in COVID-19 through initial hyperactivation of the NF-κB pathway [47]. Early type I and III IFN responses have been related to successful resolution of the infection, while late and sustained type I and III IFN responses are related to disease progression, contributing to the inflammatory storm [29,48].

Individuals who progress to the immune-mediated injury phase present decreased lymphocyte counts and significant elevation of neutrophils; the neutrophil-to-lymphocyte ratio is an early predictor of progression to severe and critical disease [42,49]. Coagulation, inflammatory and organ damage markers are significantly elevated in patients with severe and critical COVID-19: C-reactive protein, procalcitonin, ferritin, erythrocyte sedimentation rate, IL-6, IL-2, IL-7, IL-8, IL-9, IL-10, CXCL10, MCP1, MIP1A, TNF-α, bilirubin, aspartate transaminase (AST), alanine transaminase (ALT) and lactate dehydrogenase (LDH) [39,50]. Increased ferritin, neutrophil-to-lymphocyte ratio, IL-6 and D-dimer are associated to increased mortality, while decreases in B cells, T cells and NK cells were characteristically noted in severe COVID-19 at presentation [51,52]. Longitudinal comparison of lymphocyte subpopulations in patients with mild and severe disease showed marked decreases in CD3^+^, CD8^+^ and CD4^+^ T cells in severe COVID-19, while no significant differences in the trajectories of B cells and NK cells were observed in the 16 day follow-up [53]. In this same study, IL-2 and IFN-γ peaked and subsequently declined in patients with severe disease, while IL-10 and IL-6 showed a sustained elevation with respect to patients with mild disease.

Functional exhaustion of NK and CD8^+^ T cells with increased expression of NKG2, which were found to recover in convalescent patients, suggests that immune disturbance occurs early in the disease as a combination of both direct and bystander effects [54]. The finding that most patients who undergo mild-to-moderate disease produce neutralizing antibodies and specific T-cell responses [55,56], and early evidence of memory cells [57] add to the statement that an adequately mounted early immune response leads to resolution of the infection while generating specific and memory responses to the virus. However, the extent of different memory cells throughout the organs of the immune system and duration of memory remain to be determined.

Pathological studies in patients who died after developing critical disease have revealed diffuse alveolar damage, hyaline membrane, alveolar wall thickening and infiltration with macrophages, mild-to-moderate mononuclear response, viral cytopathic effects, significant diffuse hemorrhage, small vessel thrombi with surrounding CD4^+^ cells, deep venous thrombosis in some cases, and degenerated neutrophils, which could represent neutrophil extracellular traps [52,58–60]. These findings are consistent with the important immune dysregulation occurring in critical COVID-19, which together with the high pro-inflammatory cytokine levels and macrophage and monocytic infiltration resemble the macrophage activation syndrome [37], and reflect the important hypercoagulable state in critical COVID-19, which has been referred to as ‘thromboinflammation’ due to high correlation between IL-6 levels, fibrinogen and histopathologic findings [61].

The main features of hypercoagulability in critical COVID-19 are normal-to-prolonged prothrombin time and activated partial thromboplastin time, elevated D-dimer, and increased fibrinogen [62]. Tang *et al.* reported that 71.4% of nonsurvivors and 0.6% of survivors had evidence of overt deep intravascular coagulation; more patients exhibited latent deep intravascular coagulation characterized by a hypercoagulable state, as demonstrated by fibrin thrombus [63].

Pro-inflammatory cytokines are known to contribute to hypercoagulation; IL-6 contributes mainly through increased fibrinogen production, which is known to be an acute phase reactant [64]. Certain fibrinogen polymorphisms are related to greater concentrations in response to IL-6 [65], which could partially account for the varying frequencies in thrombotic complications encountered in COVID-19 patients. IL-1β and IL-8 promote rapid clot formation through increased fibrin-cross-linking and cellular clot components (more pronounced effect by IL-8), while IL-6 only mildly affects clot conformation [66].

**Figure 2.**
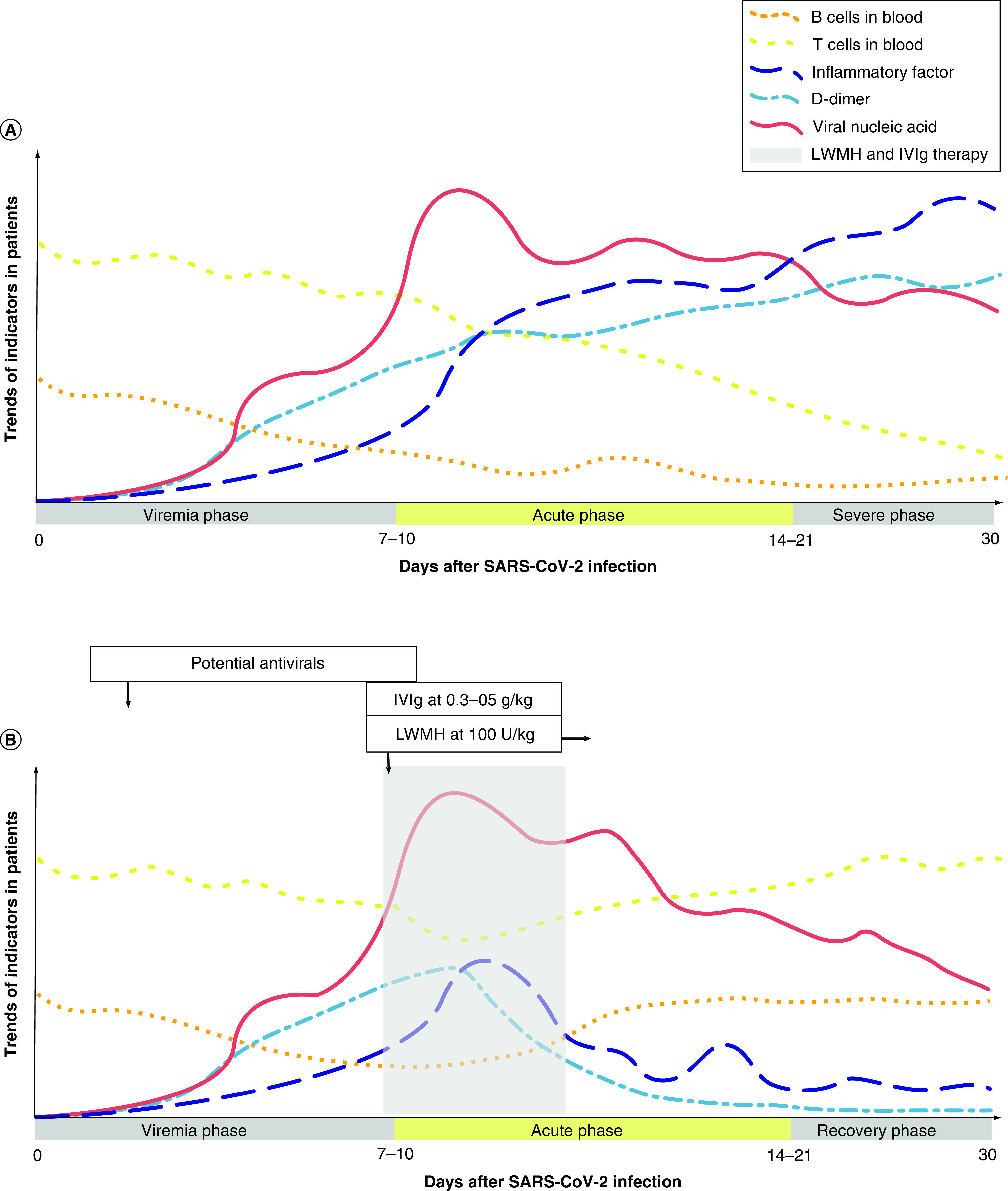
Hypothetical trajectories of selected parameters in coronavirus disease. The trend of T cells, B cells, inflammatory factors, D-dimer and viral load (y-axis) are graphed with respect to disease duration in days after SARS-CoV-2 infection (X-axis); disease course is divided into three phases (viremia, acute and severe/recovery). **(A)** Trajectories in patients reaching severe phase without specific interventions. **(B)** Trajectories in patients reaching recovery phase after LWMH and high dose IVIg therapy. The shaded areas represent the recommended intervention time for LMWH and IVIg. IVIg: Intravenous immunoglobulin; LMWH: Low-molecular-weight heparin; SARS-CoV-2: SARS coronavirus 2. Modified with permission from [13].

The clinical course of COVID-19 can be divided into three phases (viremia, acute and severe or recovery phase), under two different scenarios (with or without interventions) [13]. The first scenario (Figure 2A) reflects the natural course of patients who progress to severe and critical COVID-19. The second scenario represents the timing of interventions (i.e., antivirals, immunoglobulin and low-molecular-weight heparin) which could potentially alter the course of the disease and prevent progression (Figure 2B). Patients with significant risk factors could require more interventions than immunocompetent individuals to avoid progression to severe and critical disease.

## SARS-CoV-2 productively infects human enterocytes

Zhang and colleagues [67] noted that diarrhea accounted for a notable proportion of COVID-19 patients, ranging from 8.0 to 12.9%; high levels of hACE2 mRNA and protein expression were detected in the small intestinal enterocytes. In 2003, postmortem studies after SARS-CoV infection established the important viral tropism for intestinal cells (small and large intestine) where productive viral replication occurred, with subsequent accumulation of virions in the endoplasmic reticulum and shedding of the virus through its apical membrane. SARS-CoV-2 is known to cause less gastrointestinal manifestations than SARS-CoV, which could be partly explained by greater replication kinetics of SARS-CoV in intestinal cell lines [20]. However, SARS-CoV-2 has been shown to infect and productively replicate in human small intestinal organoids, subsequently altering gene expression, increasing cytokine production, promoting IFN-regulated genes and increasing hACE2 expression [46]. Thus, infection of intestinal cells could play an important role in promoting the inflammatory storm in COVID-19, mainly by promoting pro-inflammatory cytokines and through late and prolonged induction of IFNs.

## Gut microbiome

The trillions of microbial cells colonizing the human body were long ignored by the scientific community, but in recent years research in the field has rapidly expanded and we have learnt that microbes are a central metabolic hub, promoting in many cases physiological homeostasis and immune functions through a close symbiotic relationship with the host [68–70]. The gut microbiome has received most attention. Recently, fungi have been recognized as important components of the human microbiome, and their role in health and diseases is increasingly being studied [71,72]. In addition, during physiological states and after disturbances in the gut microbiome (i.e., single course of antibiotics), fungal species may overtake immune modulation tasks commonly done by bacteria, thereby preventing mucosal damage and *vice versa*. Viruses are also key components of the human microbiome (human virome) [73,74].

The gut microbiome spans beyond the GI tract, mediating a variety of intercommunications between the gut, enteric nervous system and the brain [75]; perturbations in the gut microbiota (dysbiosis) contribute to the initiation of pro-inflammatory signaling. For instance, dysbiosis has been found to increase translocation of bacterial lipopolysaccharide (LPS) due to increased permeability of the intestinal epithelial barrier, leading to obesity and insulin resistance [76].

Novel ways by which microbial microenvironments are shaped in the human body have started to emerge. For instance, small molecules (21–24 nucleotides) of RNA named miRNAs [77], are known to modify the gut microbiota in patients with colorectal cancer, while also altering gene expression in cancer cells, thus suggesting bilateral communication via these molecules between bacteria and human cells [78,79]. Intestinal epithelial cells have been shown to produce miRNAs which are delivered through their apical membrane to the intestinal lumen inside extracellular vesicles, and are able to enter bacteria (*Fusobacterium nucleatum* and *Escherichia coli*), altering gene expression and promoting their growth [80]. Different miRNA profiles produced by noncolitogenic and colitogenic microbiotas are associated with intestinal inflammation in the latter [81]. Interestingly, miRNAs contained in ginger exosomes after its ingestion have been shown to shape the microbiota (increased *Lactobacillaceae* and *Bacteroidales*, and diminished *Clostridiaceae*) in both humans and mice, while also altering gene expression in *Lactobacillus rhamnosus* [82]. The miRNAs contained in extracellular vesicles travelling through the bloodstream have been studied as biomarkers of inflammation and prognostic factors in patients with acute myocardial infarction [83,84], although their potential as mediators of inflammation and shapers of the microbiota at distant sites has not been studied yet.

### Dysbiosis in individuals at risk for severe & critical COVID-19

Aging and comorbidities such as hypertension, cardiovascular disease, diabetes, obesity, chronic respiratory diseases, neurologic diseases and immunosuppression are significant risk factors for severe and critical COVID-19 [40,85]. The presence of obesity-associated fatty liver disease was associated with an approximately sixfold increased risk of severe COVID-19 (unadjusted odds ratio: 5.77; 95% CI: 1.19–27.91; p = 0.029), even after adjusting for age, sex, smoking, diabetes, hypertension and dyslipidemia (adjusted odds ratio: 6.32; 95% CI: 1.16–34.54; p = 0.033) [86].

Although the precise composition of the healthy gut microbiota is not known, it is evident that microbial diversity adds to the host’s health. Obesity, hypertension and diabetes are diseases extensively associated to dysbiosis, in which increased intestinal permeability and chronic inflammation occur [87,88]. A systemic, low-grade inflammatory state is a hallmark of obesity and the metabolic syndrome. A wide range of inflammatory markers, namely C-reactive protein and pro-inflammatory cytokines are strongly associated with adiposity [89].

Multiple lines of evidence implicate gut dysbiosis as a key modulator of immune signaling in the context of metabolic diseases. LPS binds to toll-like receptors in mucosal and peripheral tissues, thereby initiating pro-inflammatory signaling [90,91]. Studies in both human and murine models have linked the obesity phenotype to endotoxemia, characterized by elevated LPS plasma levels [76,92]. Baseline levels of LPS were 20% higher in patients with obesity or glucose intolerance, whereas those with Type 2 diabetes had levels 125% higher than healthy subjects [93]. On one hand, pathogenic strains that dominate in gut dysbiosis are a rich source of LPS that could enter the circulation and initiate an immune response. On the other hand, there is strong evidence of the essential role of the gut microbiome in maintaining the integrity of the epithelial barrier; its alteration would allow increased intestinal translocation of endotoxins [94].

Microbial ecological niches are mostly limited by the external mucus layer of epithelia in the gut, interacting with the environment in the lumen and having important roles in the metabolism of components of the diet. The inner mucus layer serves as a barrier between epithelial cells and microorganisms, including potential pathogens. Symbiotic bacteria impede the proliferation of exogenous bacteria that could damage the host [95].

### Microbial interactions within the gut–lung axis

The lung microbiome has recently been recognized as a cornerstone in the physiopathology of numerous respiratory diseases [96]. The predominant bacterial phyla in lungs are the same as the ones in the gut, mainly Firmicutes and Bacteroidetes, followed by Proteobacteria and Actinobacteria [97]. From birth and throughout the entire life span, a close correlation between the composition of the gut and lung microbiome exists, suggesting a host-wide network [98]. Conversely, modifications in the lung microbiota after antimicrobial exposure may affect the gut microbiota composition [99]. Interactions between the lung microbiome and immunity are also a two-way process; major inflammatory events in the lungs can morbidly alter the composition of the lung microbiota [100].

### Intestinal permeability, translocation, inflammation & COVID-19

As mentioned in the previous section, the long-reaching immunologic impact of the gut microbiome in other organs and systems (i.e., lungs and immune system) is increasingly recognized [101]. The mesenteric lymphatic system shapes the crucial road network allowing intercommunications between the lungs and the intestine. This way, microorganisms, microbial fragments (i.e., LPS) and metabolites (i.e., short chain fatty acids) may cross the intestinal mucosal barrier and reach the lung, thereby modulating the lung immune response [102,103].

Particularly important players in this long-reaching immune interaction are gut segmented filamentous bacteria, which are commensal bacteria that colonize the ileum in most animals, including humans, and modulate the immune system [104]. Segmented filamentous bacteria regulate CD4^+^ T-cell differentiation toward the Th17 phenotype, implied in the response to pulmonary fungal infections and manifestations of autoimmune diseases in the lung [105,106]. Recently, innate lymphoid cells, which are involved in tissue repair, have been shown to be recruited from the gut to the lungs in response to inflammatory signals upon IL-25 [107]. Of note, both Th17 and innate immune cells are major sources of second order cytokines, required for effective early microbial clearance in the lungs [108].

As mentioned earlier, SARS-CoV-2 can infect cells that express high levels of hACE2 and transmembrane serine protease 2, a common feature between the lungs, esophagus, small intestine, and to a lesser extent, the large intestine. Although the specific mechanisms involved in the pathogenesis of diarrhea in COVID-19 are not entirely known, viral infection likely alters intestinal permeability, resulting in enterocyte malabsorption [86]. Intestinal inflammation, as reflected by increased calprotectin levels, correlates with the presence of diarrhea in COVID-19 [109].

Alternatively, SARS-CoV-2 could enter and shape components of the gut microbiome through glycan receptor binding via the S glycoprotein, in a similar fashion to its proven entry to common pathogens of the lungs (*Streptococcus pneumoniae* and *Pseudomonas aeruginosa*) [110]. The significance of SARS-CoV-2 entry to microorganisms of the gut and lung microbiomes remains to be studied.

Elucidating the basis of diarrhea in COVID-19 is important since patients with diarrhea had a higher need for ventilatory support (26.4 vs 8.2%; p = 0.004) and intensive care (49.0 vs 11.8%; p < 0.001), suggesting increased severity, although no correlation with the mortality rate was found [86].

### Gut microbiome & severity of infection in COVID-19

The small intestine comprises the largest organ with immune functions in humans, and we have discussed how the gut microenvironment is able to affect multiple organs and systems, including the lungs.

Researchers from Hong Kong have longitudinally characterized the bacterial [111] and fungal [112] composition of the gut microbiome in hospitalized patients through the entire spectrum of symptomatic COVID-19 (mild-to-critical), compared with non-SARS-CoV-2 pneumonia and healthy controls. Higher numbers of opportunistic bacteria (*Clostridium hathewayi*, *Actinomyces viscosus* and *Bacteroides nordii*) and fungi (*Candida albicans*, *C. auris* and *Aspergillus flavus*) were found in patients with COVID-19, while patients who received antibiotics were depleted in bacterial symbionts (*Fecalibacterium prausnitzii*, *Lachnospiraceae bacterium* 5_1_63FAA, *Eubacterium rectale*, *Ruminococcus obeum* and *Dorea formicigenerans*). Dysbiosis was found to persist throughout hospitalization, even after resolution of symptoms and a negative throat swab reverse-transcriptase polymerase chain reaction (RT-PCR) for SARS-CoV-2; fungal opportunistic pathogens also persisted (*A. flavus* and *A. niger*). Predominance of certain Firmicutes bacteria, which are negatively associated with expression of ACE2 in murine models, was also negatively correlated with severity of COVID-19. The opposite was true for other Firmicutes bacteria that are associated with greater expression of ACE2. Higher expression of hACE2 in normal human tissues has also been noted to occur under the presence of certain microbial and immunological signatures [113], which further supports the possible link between COVID-19 progression and the microbiome.

In a cross-sectional study of hospitalized patients with mild-to-severe COVID-19, gut microbial composition of patients had a low diversity compared with healthy controls, a finding similar to patients with Influenza A H1N1 infection [114]. Relative abundance of *Streptococcus* and *Escherichia/Shigella* was higher both in patients with COVID-19 and influenza. Patients with COVID-19 had predominance of *Streptococcus*, *Rothia*, *Veillonella*, *Erysipelatoclostridium* and *Actinomyces*. The authors of this study created two prediction models to distinguish COVID-19 from influenza A H1N1 infection and from healthy controls, by using gut microbial components as biomarkers, finding an overall good performance. However, this model was not validated and does not distinguish between mild and severe disease due to similar microbial compositions in both groups at admission.

Another prospective study of 15 hospitalized patients with COVID-19 found that viral infection in the GI tract occurs even in the absence of gastrointestinal symptoms and after resolution of all symptoms [115]. Interestingly, active replication of SARS-CoV-2 and transcriptional activity were correlated with shifts in the microbiota with greater abundance of opportunistic bacteria (*Collinsella aerofaciens*, *C. tanakaei*, *S. infantis* and *Morganella morganii*), while low signatures of SARS-CoV-2 infection in the gut was associated to higher presence of short-chain fatty acid producing bacteria (*Parabacteroides merdae*, *B. stercoris*, *Alistipes onderdonkii* and *L. bacterium* 1_1_57FAA).

Other studies have implied inflammation as a determinant factor of the intestinal microbiome in COVID-19, which could in turn accentuate dysregulation of the immune function: reductions in *Bifidobacterium*, *Lactobacillus* and *Eubacterium*, while significant increases in *Corynebacterium*, *Actinobacterium* and *Ruthenibacterium* (Firmicutes) were detected; fungi not commonly found in healthy subjects were also found (*Aspergillus* and *Kluyveromyces*) [116,117]. Changes in the intestinal microbiota in the context of patients with severe disease are thought to reflect the prominent inflammatory state.

Patients who develop acute respiratory distress syndrome (ARDS) have shifts in the pulmonary microbiota toward a richer composition in usual components of the gastrointestinal microbiome (*Enterobacteriaceae* and *Bacteriodetes*) [118]. Even when gastrointestinal pathogens have not been encountered in respiratory samples of patients with ARDS and SARS-CoV-2 infection, patients with COVID-19 have diminished microbial diversity in their lung microbiome, with increased abundance of opportunistic pathogens (Candida and other viruses), and transcriptional patterns that are associated with inflammation pathways [119].

## Search strategy

We searched MEDLINE and EMBASE through OVID, PubMed, BioRxiv and MedRxiv for research on COVID-19 published until 30 October 2020. We used the publicly available COVID-19 Living Evidence on COVID-19 dataset [120]. Search terms for the first search strategy related to inflammation were: (‘severe acute respiratory syndrome coronavirus 2’ [supplementary concept] OR ‘COVID-19’ [supplementary concept] OR ‘coronavirus’ OR ‘HCoV’ OR ‘nCoV’ OR ‘2019 nCoV’ OR ‘covid’ OR ‘covid19’ OR ‘severe acute respiratory syndrome coronavirus 2’ OR ‘SARS-CoV-2’ OR ‘SARS-CoV 2’ OR ‘SARS coronavirus 2’) AND (‘inflammation’ OR ‘immunity’ OR ‘immune system’ OR ‘adaptive immunity’ OR ‘innate immune system’ OR ‘cytokine storm’ OR ‘macrophage activation syndrome’ OR ‘interferon’). Search terms for the second search strategy related to the microbiome were: (‘severe acute respiratory syndrome coronavirus 2’ [supplementary concept] OR ‘COVID-19’ [supplementary concept] OR ‘coronavirus’ OR ‘HCoV’ OR ‘nCoV’ OR ‘2019 nCoV’ OR ‘covid’ OR ‘covid19’ OR ‘severe acute respiratory syndrome coronavirus 2’ OR ‘SARS-CoV-2’ OR ‘SARS-CoV 2’ OR ‘SARS coronavirus 2’) AND (‘microbiome’ OR ‘microbiota’ OR ‘human microbiome’ OR ‘lung microbiome’ OR ‘respiratory microbiome’ OR ‘gastrointestinal microbiome’ OR ‘gut microbiome’). Studies were chosen regardless of language, provided an abstract in English was available, and if considered relevant by consensus of at least two authors.

For studies published prior to 2020, no specific search strategy was conducted and references were chosen at the authors’ best judgment to provide educational context to readers, and may thus be biased toward the authors’ opinions in this perspective article.

## Conclusion

Knowledge on the mechanisms at the molecular, cellular and systems levels by which SARS-CoV-2 causes disease in humans have been studied and communicated in an incredibly expedite manner never seen before. SARS-CoV-2 enters a wide variety of human cells in an extraordinarily efficient way which allows the virus to evade the immune system initially, thereby impeding robust type I and III IFN responses much needed to eliminate the virus. Dysregulation of RAAS and late type I and III IFN overactivation contribute to progression of the disease in susceptible individuals, although other mechanisms leading to severe and critical COVID-19 are most likely uncovered at this moment. Potential infection of cells of the immune system and direct damage to these cells by SARS-CoV-2 remains to be studied to understand how the immune response could be directly affected by the virus.

We have discussed some ways by which the microbiota could possibly promote inflammation in individuals with COVID-19. We have mentioned bacterial translocation and the presence of LPS in the bloodstream as a potential promoter of inflammation, which could contribute in a similar way to the inflammatory storm which occurs in sepsis. miRNAs are also potential promoters of systemic inflammation which could be acting in different ways. For example, established dysbiosis in patients with chronic diseases could be the set point allocating these patients at increased risk of developing greater inflammation. Studying dietary and lifestyle habits in persons with comorbidities before COVID-19 infection and looking for differences in these groups could be an interesting way of establishing this connection. Cohorts of patients with chronic diseases in whom the microbiota composition has been prospectively studied could serve as the perfect group to study how dysbiosis affects predisposition to COVID-19 severity and outcomes.

The gut and lungs are robustly intercommunicated through pathways such as the mesenteric lymphatic system, although the bloodstream could also serve as another highway communicating the lungs and gut through small molecules such as bacterial metabolites or miRNAs, which are likely to be able to travel long distances in the body since they are contained inside extracellular vesicles. What happens in the lung can have consequences in the gut and *vice versa*, while intercommunication through the immune system is undoubted.

Interestingly, SARS-CoV-2 has been shown to enter bacteria through rhamnosylated epitopes which are present in some pathogenic bacteria. Exploring the expression of these epitopes in components of both healthy and dysbiotic microbiomes could be the first step toward understanding if SARS-CoV-2 is able to shape the microbiota by directly interacting with their components. The same could apply to fungal components of the microbiome.

To date, only the microbiota (taxonomy of microbial components) has been studied in the context of COVID-19. Studies aiming to describe the metagenomics and transcriptomics of microbial components in the context of COVID-19 will allow to start characterizing the extent to which the microbiome affects or is affected by COVID-19.

Finally, answering these questions could allow the scientific community to develop ingenious ways to ameliorate disproportionate inflammation, prevent disease progression or improve patient outcomes.

## Future perspective

A great number of scientists from multiple backgrounds have united efforts and redirected their attention toward rapid characterization of SARS-CoV-2 and the disease it causes in humans (COVID-19). This is unprecedented, since no biologic phenomena had ever drawn this amount of attention. Discoveries in this topic have occurred in an impressively accelerated way, and the pace at which breakthroughs are occurring suggests that it will not be long until we have precise characterizations of the virus, its pathophysiology and available therapeutics, including vaccines, to combat this pandemic. Characterization of the interplays between the lungs, gut, immune system and the human microbiome will aid to have a greater understanding of how these elements could determine disease progression or modulate responses to infection. This will open new venues toward developing innovative interventions for COVID-19.

**Executive summary**

- Coronavirus disease (COVID-19), the pandemic caused by the SARS coronavirus 2 (SARS-CoV-2), has challenged current societies due to rapid spread and lack of therapeutics.

**SARS-CoV-2 utilizes efficient mechanisms to establish infection & evade the innate immune response**

- The mechanisms involved include antigen hiding, pre-activation of its spike protein and avid recognition of its receptor, the human angiotensin-converting enzyme 2. This allows the virus to initially prevent adequate interferon (IFN) responses.

**Immune-mediated injury is the predominant pathophysiological driver in severe & critical COVID-19**

- Early IFN responses have been related to successful resolution of the infection, whereas late and sustained IFN responses are related to disease progression.
- Unregulated angiotensin II could promote inflammation pathways, especially in individuals with diseases involving alterations in the renin–angiotensin–aldosterone system.
- Progressive increases in inflammation and hypercoagulability are two main hallmarks of severe and critical COVID-19.
- Clinical, pathologic and experimental studies have revealed the central role of inflammation in COVID-19 pathophysiology.
- Early interventions in patients with risk factors could stop progression to severe and critical COVID-19.

**SARS-CoV-2 productively infects human enterocytes**

- SARS-CoV-2 productively infects human enterocytes, altering gene expression, cytokine production, IFN pathways and human angiotensin-converting enzyme 2 expression.

**Gut microbiome**

- The components of the human microbiome comprise different microorganisms (i.e., bacteria, viruses and fungi).
- miRNAs are 21–24 RNA molecules frequently encountered inside extracellular vesicles. miRNAs produced by the intestinal epithelium and from diet are able to shape the gut microbiome, while certain miRNA profiles promote shifting to a microbiome associated with decreased or increased intestinal inflammation.

**Microbial interactions within the gut–lung axis**

- The gut–lung axis results from complex interactions between the different microbial components of both the gut and lung niches, and the immune system. Events occurring in the lungs are able to alter the gut and *vice versa*.

**Intestinal permeability, translocation, inflammation & COVID-19**

- Microorganisms, microbial fragments (i.e., lipopolysaccharide) and metabolites (i.e., short-chain fatty acids) may cross the intestinal mucosal barrier and reach the lung through the mesenteric lymphatic system and bloodstream.
- Diarrhea is an important manifestation of COVID-19, which correlates with inflammation and disease severity. This symptom is likely a result of malabsorption, although SARS-CoV-2 is able to enter microorganisms with rhamnosylated epitopes, which leaves an open question for possible direct effects in microbial components of the microbiota.

**Gut microbiome & severity of infection in COVID-19**

- Few studies have investigated the role of the microbiome in COVID-19. In patients without diarrhea, dysbiosis was related to disease severity.
- In acute respiratory distress syndrome, the lung microbiota shifts toward a richer composition in habitual intestinal microorganisms.
- Studies with translational approaches are needed to better understand COVID-19 and possible interventions.
